# Sol/gel transition of oil/water microemulsions controlled by surface grafted triblock copolymer dodecyl–PEO_227_–dodecyl: molecular dynamics simulations with experimentally validated interaction potential

**DOI:** 10.1039/d1ra02649f

**Published:** 2021-06-11

**Authors:** M. Khatouri, M. Lemaalem, R. Ahfir, S. El Khaoui, A. Derouiche, M. Filali

**Affiliations:** Laboratoire de Physique Appliquée, Informatique et Statistique (LPAIS) Sidi Mohamed Ben Abdellah University, Faculty of Sciences Dhar El Mahraz BP 1796, Atlas Fes Morocco; Laboratoire de Physique des Polymères et Phénomènes Critiques Sciences, Faculty Ben M'Sik, Hassan II University P.O. Box 7955 Casablanca Morocco mohammedlemaalem@gmail.com

## Abstract

We studied a large range of identical spherical oil/water microemulsion (O/W-MI) volume fractions. The O/W-MIs are stabilized by cetylpyridinium chloride ionic surfactant (CpCl) and octanol cosurfactant and dispersed in salt water. We grafted different numbers of dodecyl–(polyEthylene oxide)_227_–dodecyl triblock copolymer that we note (*n*(D–PEO_227_–D)), where *n* varies from 0 to 12. We accomplished the grafting process by replacing a small amount of CpCl and octanol with the appropriate *n*(D–PEO_227_–D). The aim is to determine the interaction/structure relationship of the covered microemulsions. Precisely, we are interested in a quantitative investigation of the influence of volume fraction *Φ*, temperature (*T*), and *n*(D–PEO_227_–D) on the microemulsion sol/gel transition. To this end, we first study the uncoated microemulsion structure depending only on *Φ*. Second, we determine the coated microemulsions structure as a function of *n*(D–PEO_227_–D) for different *Φ*. Third, we examine the effect of temperature on the uncoated and coated microemulsion. We show that the sol/gel transition is controlled by the three main parameters, *Φ*, *T*, and *n*(D–PEO_227_–D). Accordingly, the uncoated microemulsion sol/gel transition, at ambient temperature, occurred for *Φ* ≃ 33.65%. By increasing *Φ*, the O/W-MIs show a glass state, which occurs, along with the gel state, at *Φ* ≃ 37% and arises clearly at *Φ* ≃ 60%. The coated O/W-MI sol/gel transition is found to be linearly dependent on *n*(D–PEO_227_–D) and takes place for *Φ* ≃ 26.5% for *n*(D–PEO_227_–D) = 12. Ordinarily, the decrease in temperature leads to gel formation of microemulsions for low *Φ*. Additionally, in this work, we found that the gelation temperature increases linearly with *n*(D–PEO_227_–D). Thus, the parameter *n*(D–PEO_227_–D) can control the sol/gel transition of the O/W-MIs at ambient temperature and moderate *Φ*.

## Abstract 2

We studied the sol/gel transition of oil/water microemulsion (O/W-MI) covered with dodecyl–(polyEthylene oxide)_227_–dodecyl triblock copolymer.

## Graphical abstract

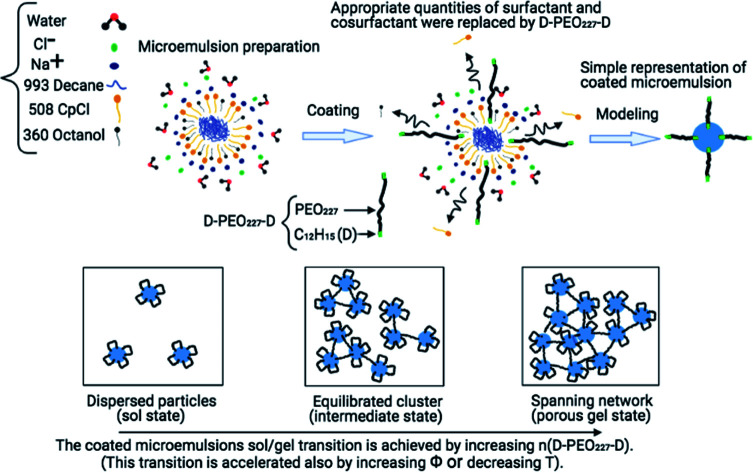

## 1 Introduction

Microemulsions as drug carriers for drug enhancement, protection, and diffusion have contributed to developing the field of drug delivery.^1^ Methods such as compression, spray, dip coating, and encapsulation, incorporating bioactive agents with polymers, have been employed in pharmaceutical manufacturing for over 60 years.^2–5^ These polymers included cellulose and chitin derivatives, poly(ethylene glycol) PEG, and poly(ethylene oxide) PEO.^6–9^ From a drug delivery perspective, polymeric devices are classified into diffusion-controlled, solvent-activated,^10^ chemically-controlled, or external triggers.^11^

**Fig. 1:**
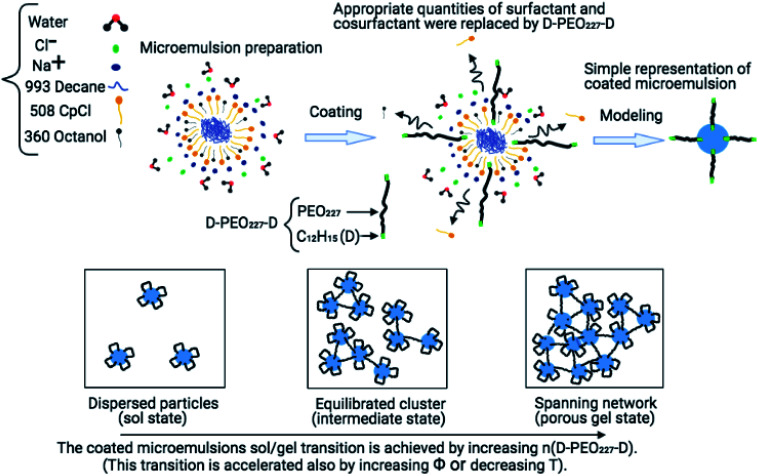
Schematic illustrations represent the main steps and findings of the conducted studies.

Conventionally, colloidal suspensions which undergo self-assembly and gelation are widely used as carriers in drug delivery. The gelation is controlled by the manipulation of attractions, such as electrostatic attraction,^12^ depletion,^13–15^ polymer bridging effect,^16,17^ and van der Waals interactions.^18,19^ In a recent report, we conducted a study concerning the control of steric repulsions by adding hydrophobic PEO_227_–dodecyl polymers to induce microemulsion flocculation.^20^ Also, the addition of electrolytes in colloidal suspensions is reported to reduce the range of repulsion, resulting in aggregation due to van der Waals or other attractions.^21^ However, this method requires control of salts concentrations, which may be difficult for materials processing. Therefore, the addition of polymers with two hydrophobic ends, which can assure the bridging between microemulsions, can modulate attractive interactions effectively and independently of salts concentrations.^22^Fig. 1 depicts a schematic representation of the chemical composition of the coated microemulsions. The grafted polymers can bridge two or more microemulsions or decorate one microemulsion. Then the added polymers control the microemulsions distribution and lead to the formation of different physical states.

Gelation is the process by which a sol becomes a gel.^23^ In simple terms, arguably, nanoparticles in dispersions collide and stick to each other even at low colloidal particle volume fractions,^24,25^ creating a porous solid network, which encloses the liquid phase. The gel is a specific type of colloid. It can be defined either as a deformable and elastic solid, formed by an assembly of colloidal particles, droplets, or connected macromolecules to each other or as a semi-solid liquid.^26–28^ The gel consists of at least two components, the first is a dispersed or solubilized forming the gel network called the gelling agent, and the second is the solvent, a compound with liquid-like behavior.^29,30^ We recall that it is possible to make gels from dispersed microemulsions by controlling three parameters: the attractive interactions, the volume fraction of the particles, and the temperature.^31^

The microemulsions sol–gel transition is low-cost and conductible at living temperatures.^32^ Besides, it is controlled by the chemical composition of the microemulsions cover. Even small graft polymers quantities can be introduced in the sol to form a gel.^33^ From sol to gel, there are several physical states.^34^ Thus, several derived microemulsions systems can found their applications in diverse areas.

Covered microemulsions that are suspended in a liquid intrinsically interacts *via* attractive and repulsive interactions. The attractive ones encompass the van der Waals short-ranged attractive interactions and the bridging effect induced by the grafted polymers, at least one polymer chain adsorbs on two or more microemulsions simultaneously.^33^ Thus, the polymer causing a strong attraction interaction, independently of the solvent quality. If the microemulsions are overcoated, the dominance of such interactions makes the microemulsions inherently unstable.

The repulsive interactions can be electrostatic, steric, or both of them. The electrostatic repulsion is resulting from the number of charges per microemulsion.^20^ The steric interaction results from the cover polymers excluded volume.^20^ The strength of the steric repulsion interaction introduced by the graft polymers is significant for the good-solvent case, and it is attributed to the osmotic interactions between the polymers segments surrounding each microemulsion.^35^ However, this interaction weakens in the case of the bad-solvent condition. The repulsive interactions counterbalance the attractive interactions and prevent microemulsions from colliding and sticking together. Thus, they are necessary for the microemulsions stabilization.

From the literature, Molecular Dynamics simulations (MD) were used in several works to study the colloidal particle gel characteristics. In this context, Ethayaraja Mani *et al.* used molecular dynamics simulation to study the phase behavior of colloids interacting *via* short-range attraction and long-range repulsion. They studied the effect of the strength of attractive interaction on the phase behavior at different colloid densities. They presented the non-equilibrium and equilibrium cluster phases with well-defined internal structures and noticed that the equilibrium cluster phase contains small and compact clusters, while the non-equilibrium ones are found large and elongated.^36^ A Coniglio *et al.* presented MD simulations of uncoated colloidal particles interacting *via* the Derjaguin–Landau–Verwey–Overbeek (DLVO) potential. They have proved that colloids exhibit a gel state at room temperature for high fractions. Besides, structural arrest with gel characteristics was found at low volume fraction and low temperature.^37^

In this paper, we discussed the interaction/structure relationship of microemulsions covered triblock copolymer dodecyl–PEO_227_–dodecyl using Molecular Dynamics simulations (MD) as the prime method. The interaction potential introduced in the MD simulations is verified by comparing the structure factors *S*(*q*) obtained *via* the Fourier transform of the radial-distribution-function, calculated using MD simulations with the adjustable parameters listed in Table 1. The study was conducted for different volume fractions *Φ*, temperatures, and the number of the surface grafted polymers *n*(D–PEO_227_–D), with a particular emphasis on their effect on the sol/gel transition.

**Table 1:** Interaction potential parameters

	*R*	*A*_H_	*κ* ^−1^	*Z*_eff_	*l*_B_	*A*_steric_	*λ*_steric_	*A*_bridging_	*λ*_bridging_
Bare microemulsions	62 Å	1.1*k*_B_*T*	6.77 Å	130	7.18 Å	—	—	—	—
Microemulsions + 4(D–PEO_227_–D)	62 Å	1.1*k*_B_*T*	6.77 Å	130	7.18 Å	2.6*k*_B_*T*	45 Å	−0.53*k*_B_*T*	90.71 Å
Microemulsions + 8(D–PEO_227_–D)	62 Å	1.1*k*_B_*T*	6.77 Å	130	7.18 Å	5.2*k*_B_*T*	45 Å	−1.05*k*_B_*T*	90.71 Å
Microemulsions + 12(D–PEO_227_–D)	62 Å	1.1*k*_B_*T*	6.77 Å	130	7.18 Å	7.8*k*_B_*T*	45 Å	−1.10*k*_B_*T*	90.71 Å

## 2 Materials and methods

### 2.1 Materials

Cetylpyridinium chloride [H_3_C–(CH_2_)_15_]–C_5_H_5_N + Cl^−^ (CpCl) was purchased from Fluca and was purified by successive recrystallization in water and in acetone. The octanol [H_3_C–(CH_2_)_7_]–OH and decane [H_3_C–(CH_2_)_8_CH_3_] were obtained from Fluca and were used as received. All samples were prepared by weight in brine or deuterated brine. Brine (0.2 M NaCl) was prepared with triply distilled water or deuterated water from Material Safety Data Sheet (MSDS).

### 2.2 Microemulsions preparation

The studied microemulsions are thermodynamically stable microspheres. They are composed of decane surrounded by CpCl surfactant and octanol co-surfactant tiny films (hydrophobic part) dispersed in saltwater. We recall that there is a limit to the formation of microemulsions. This limit is controlled by the ratio in weight of decane to the surfactants. This ratio indicates a limit value, above which the microemulsions are saturated with decane and coexist with its excess. Before this limit, the microemulsions are spherically shaped.^38,39^ Their size is corresponding to the spontaneous curvature radius of the surfactants film. We note that there is no condition regarding the ratio of saltwater to decane or surfactant. Thus the microemulsions can be diluted over a wide range of concentrations (1 to 20% by weight). The weight ratio of octanol to CpCl is 0.25. Then decane is added up to the emulsification failure limit. Thus, a value slightly lower than this limit is adopted. The ratio by weight of decane to surfactants molecules (CpCl + octanol) is, in what follows, equal to 0.62. Later, we add a telechelic polymer, the dodecyl–PEO_227_–dodecyl, that we note (D–PEO_227_–D), to the decane/water microemulsions. Where the PEO_227_ is a polyethylene oxide of formula HO−(CH_2_–CH_2_–O)_227_–CH_2_–CH_2_–OH to which an aliphatic chain, CH_3_−(CH_2_)_11_–NCO, and therefore hydrophobic, is grafted by making an NCO isocyanate function connected to a dodecyl (C_12_H_25_) aliphatic chain act on the alcohol functions located at the ends of the polyethylene oxide. The graft isocyanate function gives each of the PEO_227_ ends a urethane NH(CO)O function, which leads to the junction between the PEO_227_ and the hydrophobic ends. In this case, the D–PEO_227_–D copolymer can bridge two or more microemulsions since each dodecyl can integrate its hydrophobic part. Thus, the D–PEO_227_–D is an amphipathic macromolecule.

The overall composition is determined to obtain a constant volume fraction of the microemulsions hydrophobic parts (decane plus the CpCl and octanol hydrophobic parts plus the alkyl chains of D–PEO_227_–D), gradually increasing the number of adsorbed alkyl chains. This process is conducted by replacing a small amount of CpCl + Octanol with the appropriate number of D–PEO_227_–D chains per microemulsions *n*(D–PEO_227_–D). It is assumed that the radius of the hydrophobic part of the microemulsions (decane + adjacent surfactants) does not change with the increasing substitution of the surfactant by the added *n*(D–PEO_227_–D). The samples are shaken to accelerate the surfactants and co-surfactants self-assembly (phase separation between the hydrophobic and the hydrophilic parts) and therefore accelerated formulation of the microemulsions.

### 2.3 Small angle neutron scattering (SANS) measurements

**Fig. 2:**
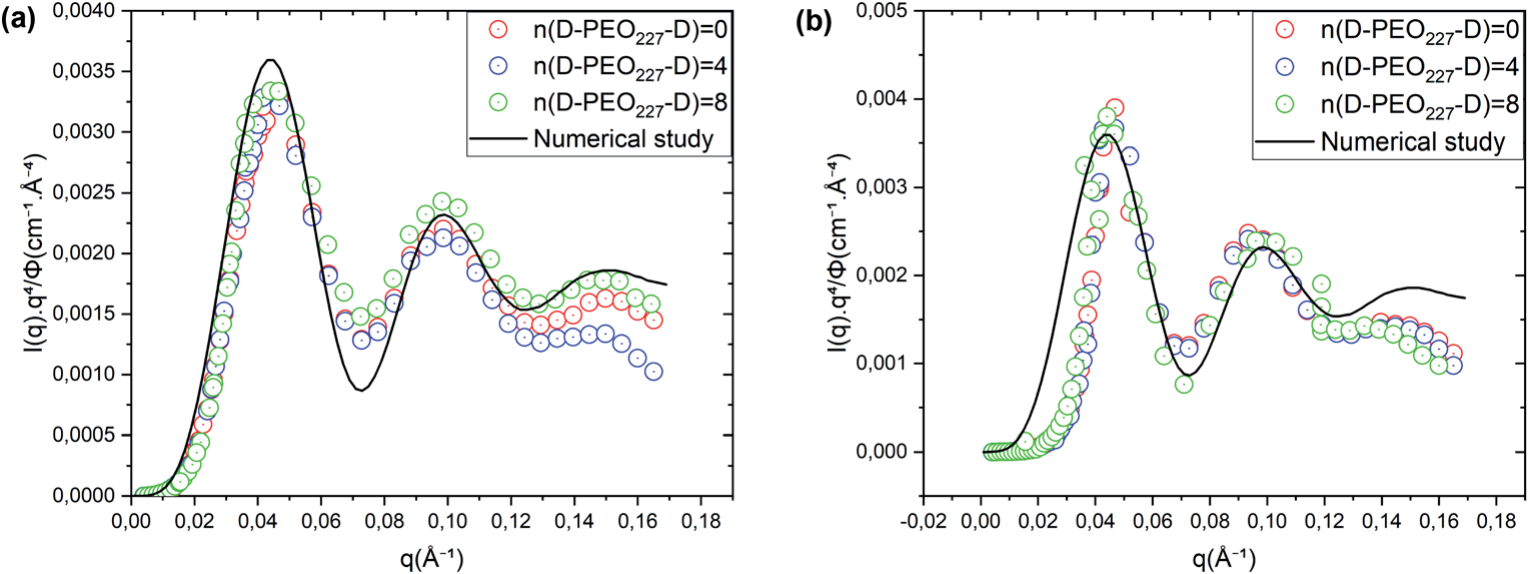
Renormalized spectra in Porod representation (*q*^4^ × *I*(*q*)) for *Φ* = 6.98% (a) and *Φ* = 26.5% (b). The solid line in black is the computed spectra, using formula (10), with *R̄* = 62 Å and Δ*R* = 6.2 Å.

The SANS experiments have been performed at LLB-Saclay on the spectrometer PACE. We covered a large range of scattering vectors from *q* = 0.004 Å^−1^ to *q* = 0.16 Å^−1^. The scattering data are put on an absolute scale by using water as the standard. The intensities are obtained in absolute units (cm^−1^) with an accuracy of less than 10%. The neutrons are scattered by the microemulsions and the scattered intensity is described by the usual formula:1
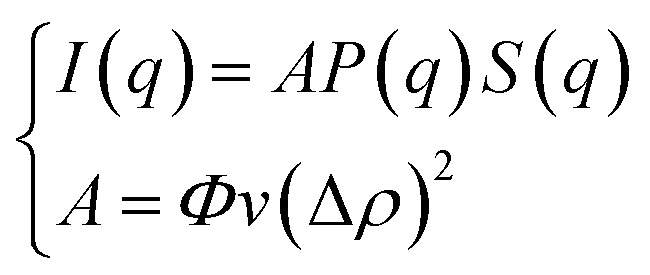
where *S*(*q*) the structure factor, *q* (Å^−1^) the scattering vector, *Φ* the microemulsions volume fraction, *v* (cm^3^) the microemulsions dry volume, Δ*ρ* = 6.83 × 10^10^ cm^−2^ the contrast, *i.e.*, the difference between the microemulsions and the solvent scattering length density, and *P*(*q*) the microemulsions form factor, such as *P*(*q* → 0) = 1. At the limit of high *q* values, the structure factor *S*(*q* → ∞) = 1. Thus, the scattered intensity is related to the microemulsions form factor. We use the Porod representation, (*q*^4^ × *I*(*q*)) as a function of *q*, to amplifies the *P*(*q*) oscillations. The formed microemulsions are spherically shaped, with a low size polydispersity. The latter is described by a Gaussian distribution with a mean radius *R̄* = 62 Å, and a standard deviation, Δ*R* = 6.2 Å as presented in Fig. 2. Thus, their average form factor is:2
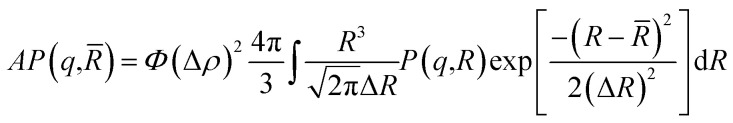
with the form factor of monodisperse microemulsions of radius *R*3
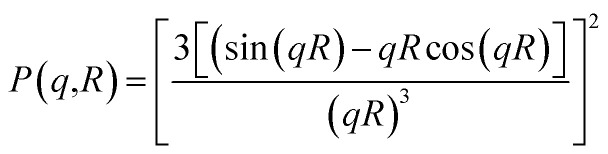


The first two maximum and the first minimum of the above formula are therefore:
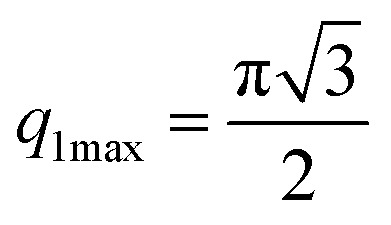
, *q*_2max_ = 2π, 
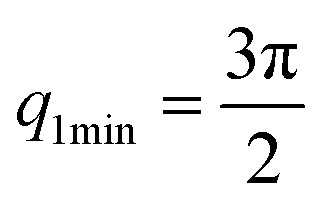


### 2.4 Molecular dynamics simulation (MD)

Molecular dynamics is a method that consists of following the evolution of interacting particles over time. This simulation is applied to study the structural and thermodynamic properties of the system, and to understand the experimental results, provided that the interaction potential between the system particles is known.

The flocculation of colloidal suspensions is resulting from attractive van der Waals interactions and the Brownian motion.^40,41^ Besides, kinetic stability is resulting from repulsive interactions. There are essentially two types of repulsive interactions. The first type, the repulsion results from electrostatic forces exerted between charged colloidal surfaces and which are screened by counter-ions that revolve around the colloid surface. From the literature, the exact interaction potential between two charged colloids was first calculated by Derjaguin and Landau^42^ then by Verwey and Overbeek,^43^ as a part of the DLVO model (Derjaguin, Landau, Verwey, Overbeek). The second type consists of colloids covered by neutral polymers. Under the conditions of good-solvent, the adsorbed polymers represent a sufficient steric barrier, which maintains the dispersity of the colloids.^44^ If polymers can bridge the microemulsions, they produce an attractive interaction.

In the present work, molecular dynamics simulation was used as the main method to determine the physical properties of a system consisting of ionic microemulsion particles covered with D–PEO_227_–D triblock copolymer. The interaction between the ionic microemulsion particles is modeled by a DLVO potential,^45^ and has an effective interaction potential, which is the sum of a hard-sphere repulsion potential *U*_HS_, a van der Waals attractive term *U*_VW_. In the case of charged microemulsions, a Yukawa type screened coulombic interaction potential *U*_coul_ is added. Besides, the addition of *n*(D–PEO_227_–D) chains on microemulsions has two possible configurations. The first one is when the droplets are far from each other the polymer chains are too short to bridge between the microemulsions: a chain with a sticker in a given microemulsion is forced to make a loop so that the second sticker adsorbs on the same microemulsion and the polymers contribute by a steric interaction *U*_steric_. The second one, if the droplets are close together, the looped conformations are still accessible. Besides, the bridging conformations are also accessible. In this case, we have seen an attractive additional contribution related to the bridging of the microemulsions by the D–PEO_227_–D chains *U*_bridging_. Thus, the total pair interaction potential is expressed as:

4 *U*(*r*) = *U*_HS_ + *U*_VW_ + *U*_coul_ + *U*_steric_ + *U*_bridging_

with:5
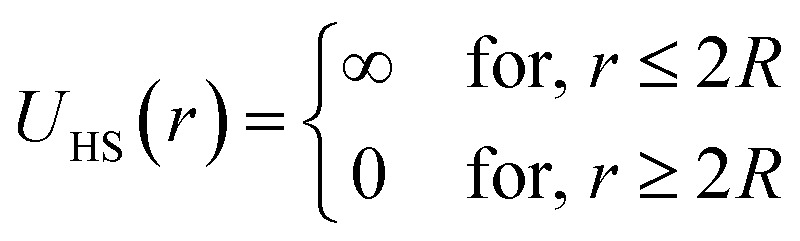
6
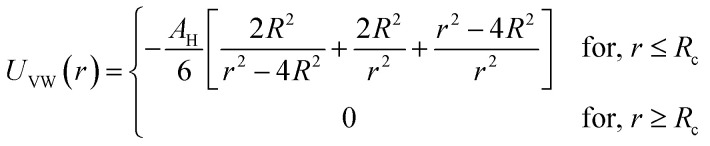
where *r* is the centre-to-centre distance and *R* is the microemulsion radius. *A*_H_ is the Hamaker constant. *R*_c_ is a cut off distance, of the interaction potential.7
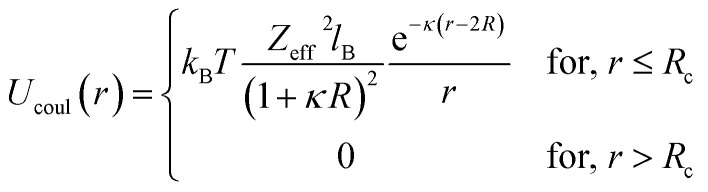
where *l*_B_ is the Bjerrum length, 
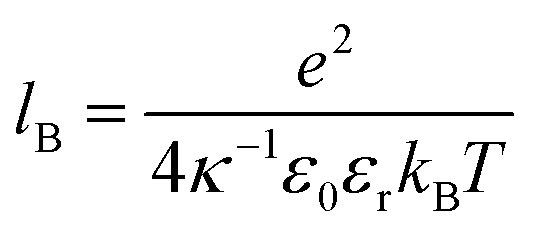
, with *ε*_r_ the dielectric constant of water. *T* is the temperature, and *κ*^−1^ represents the Debye length, which corresponds to the concentration of small ions added to the solution. *Z*_eff_ is the number of effective elementary charges (*e*) per microemulsion.8
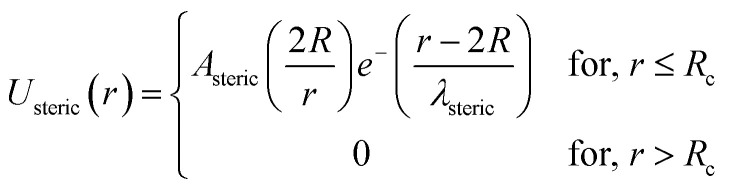
with *A*_steric_, the contact potential and *λ*_steric_, the interaction range which is assumed to be of the order of magnitude of the PEO_227_ radius of gyration, defined by *R*_g_ = 0.0215 × *M*^0.58^ nm = 44.96 Å, where *M* is the PEO_227_ molar mass in g mol^−1^, such as *M* = 44.05 × 227 + 18.02 g mol^−1^.^46^9
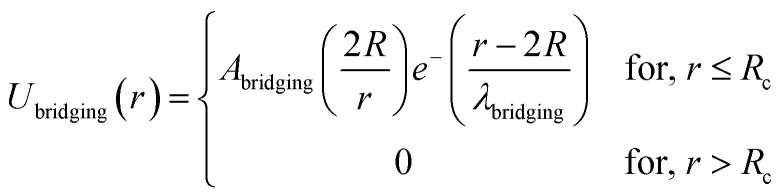
with *A*_bridging_, the contact potential and *λ*_bridging_, the range of the interaction which should be of the order of magnitude of the PEO_227_ Flory radius, defined by *R*_F_ = *a* × *N*_p_^3/5^ = 90.71 Å,^8^ where *a* = 0.35 nm, the oxyethylene radius measured by K. Hristova and D. Needham,^47^ and *N*_p_ = 227, the degree of polymerization of the PEO_227_ representing the number of PEO per a PEO_227_ polymer chain.

We note that the potential parameters *λ*_steric_ and *λ*_bridging_ are determined theoretically and are unchanged for the studied range of grafting density. In contrast, the parameters *A*_steric_ and *A*_bridging_ strongly depend to the variation of the added polymers per microemulsion and are numerically adjusted to reproduce the SANS structure factor *S*(*q*) using the HNC numerical method, before introducing them in the MD simulation. The basic of HNC method is previously discussed by Lemaalem *et al.*^45^

**Fig. 3:**
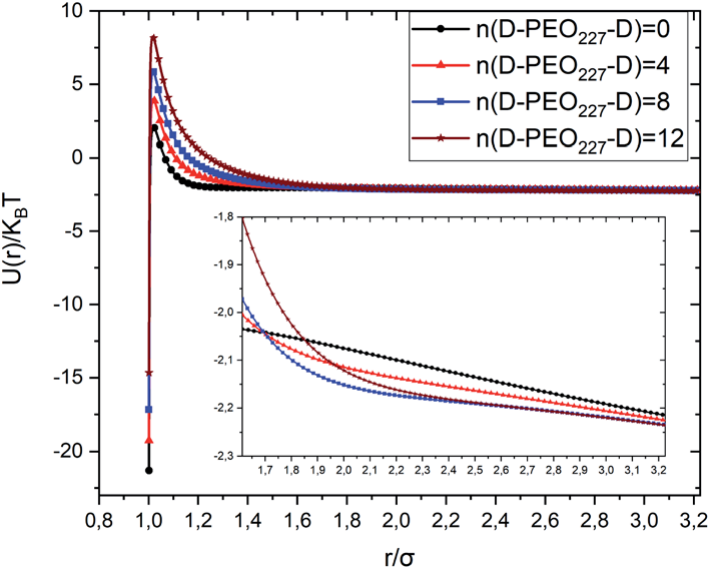
Dimensionless pair-potential interaction between microemulsions, *U*(*r*)/*k*_B_*T*, *versus* the dimensionless distance *r*/*σ* (*σ* = 2*R*).

Fig. 3 depicts the total interaction potential for several values of *n*(D–PEO_227_–D). At short range-distances (2*R* + *R*_g_ < *r* < 2*R* + *R*_F_), the cover polymers add an important repulsion part to the electrostatic contribution. Besides, at intermediate distances (2*R* + *R*_g_ < *r* < 2*R* + *R*_F_), the suspected bridging effect adds an attractive component to the vDW contribution.

We performed molecular dynamic simulations (MD) in the *NVT* statistical ensemble to mimic the experimental conditions, using the LAMMPS simulation package.^48^ The number of simulated microemulsions is of *N* = 10^6^, the volume of the simulation box (*V*) is changed to study the microemulsions in different volume fractions. The initial configuration is randomly generated. Then, it is sufficiently equilibrated for 10^6^ time-step, with a time-step δt* = 0.01 in the *NVT* ensemble before producing the results. In such a manner, different simulations with the same volume fraction, temperature, and a fixed number of added polymers, regardless of the number of particles, must lead to the same thermodynamics equilibrium. The temperature (*T*) is set to the ambiante temperature *T*_a_, *T* = *T*_a_ = 25 °C. The surface effects are removed using the periodic boundary conditions and the reduced units are used to optimize the MD calculation time, the time is reduced by 
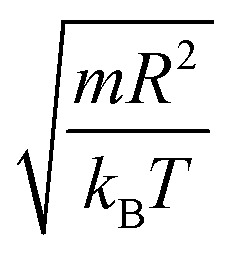
, the energies are reduced by *k*_B_*T*, the distances are reduced by the O/W-MI radius *R*, and the temperature is reduced by the room temperature *T*_a_.^48,49^

We analyzed the O/W-MIs structural properties using the radial distribution function *g*(*r*). The *g*(*r*) presents the probability of finding microemulsions located at each separation distance *r* around a central one. Thus, the *g*(*r*) provides a clear description of the O/W-MIs distribution. Besides, it is related to the structure factor *S*(*q*) available from SANS experiments.10
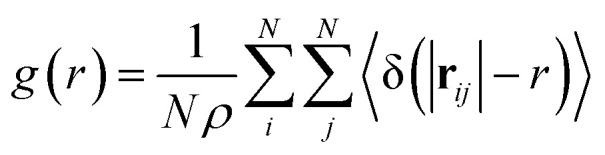
where *N* is the number of O/W-MIs, |**r**_*ij*_| is the separation distance magnitude between two different O/W-MIs (*i* and *j*), and *ρ* = *N*/*V* is the O/W-MIs number density.11
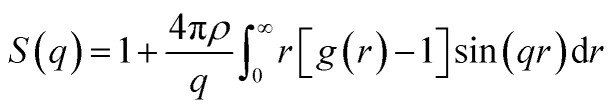
where *q* is the momentum transfer vector magnitude.

**Fig. 4:**
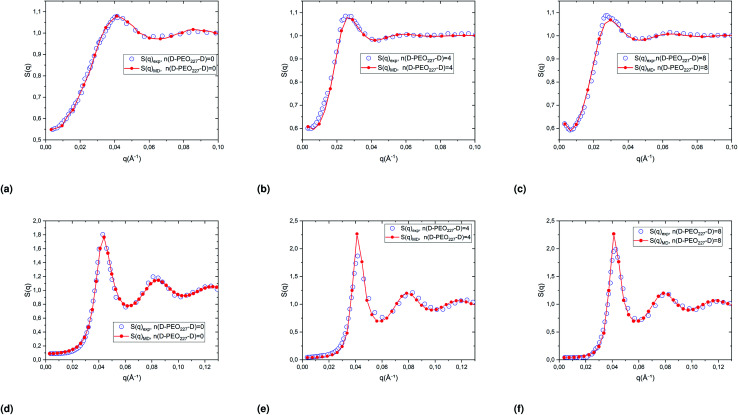
Structure factors of the studied microemulsions with two different number of added D–PEO_227_–D and two different volume fractions, (a)–(c) *Φ* = 6.98%, (d)–(f) *Φ* = 26.5%. The curves are obtained from the MD simulations, using the interaction potential parameters presented in Table 1, and SANS experiments.

Fig. 4 represents a comparison of the structure factors obtained from SANS experiments *S*_SANS_(*q*), and those obtained from molecular dynamics simulation *S*_MD_(*q*), using the interaction-potential parameters presented in Table 1. The MD results and the SANS findings are an excellent agreement, proving that the proposed potential model is satisfactory to study microemulsions covered with a different number of added dodecyl–PEO_227_–dodecyl *n*(D–PEO_227_–D) for different volume fractions and amount of added polymers. For high volume fractions, we notice that the principal peak becomes narrow and more pronounced with increasing *n*(D–PEO_227_–D) but have almost the same *q*_max_. For low volume fractions, the heights of the main peaks are almost the same. But their positions *q*_max_ are shifted to lower values when *n*(D–PEO_227_–D) increases.

We note that *q* is inversely proportional to the average distance between the microemulsions. Thus, for a small volume fraction, the addition of D–PEO–D is supposed to induce a repulsion between microemulsions. In contrast, for high volume fractions, the polymers favor the attraction.

## 3 Results

### 3.1 Effect of the volume fraction on the microdistribution of decane/water microemulsions

The structure factors obtained from MD simulations and SANS experiments are compared in Fig. 4 to verify the effective interaction potential form and parameters representing the direct interaction between the dispersed decane/water microemulsions. Thus, the structural properties are analyzed using the radial distribution function *g*(*r*), obtained from extended MD simulations, which refers to us as the probability of finding neighbors particles around a central one.

We note that, for the dispersed microemulsions, the radial distribution function is characterized by a periodicity of maxima and minima peaks. The maxima indicate the most probable distance between the neighbor particles surrounding a central particle. While the minima correspond to a high-energy area of space that prevents particles from existence in these distance ranges.

**Fig. 5:**
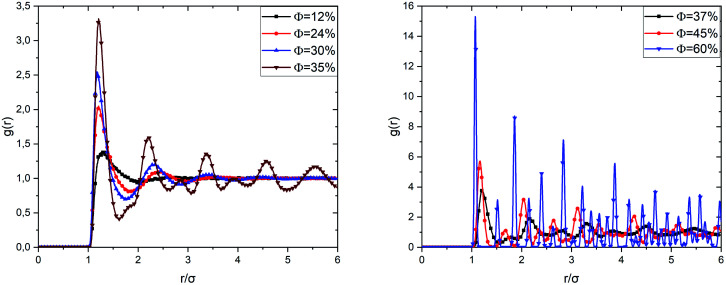
Radial-distribution-function *g*(*r*) as a function of the volume fraction *Φ*.

Fig. 5 shows the radial distribution functions of the uncoated microemulsions, calculated from the Molecular Dynamics simulation (MD) at ambient temperature (*T* = 298 K), for different volume fractions. For low and medium volume fractions (*Φ* ≤ 30%), the system is in a sol state, which is visible in the radial distribution functions. We observe that in the very diluted case (*Φ* = 12%), the correlation function shows a single wide-peak, which is due to the interactions between the first close neighbors. The peak corresponds to an average distance between the microemulsions of the order of 1.27*σ*. This distance range reveals that at low *Φ* the microemulsions are dispersed, and the electrostatic repulsion is dominant.

When the volume fraction increases from *Φ* = 12% to *Φ* = 35%, we notice that the correlation peak shifts towards small distances (from 1.27*σ* to 1.21*σ*), becomes more intense and narrower. A second peak appears due to the interactions between a considered microemulsion and its second neighbors. This large-peak indicates that the microemulsions are distributed randomly over space. We notice that the first peak height increases with increasing the volume fraction. We have attributed this increase to progressive domination of the van der Waals interaction potential since the separation distance between the microemulsions shifts to the smallest values. We also observed a sub-peak appearing before the second correlation peak for *Φ* = 35%. This sub-peak can be considered as an indication of the transition from the liquid to the gel state. This phase is called gelation. The simple gelling scheme is that the microemulsion nanodroplets collide with each other, forming a continuous porous solid network, which encloses the liquid phase.^50^ By increasing *Φ*, a highly disordered amorphous structure is observed at *Φ* = 37%. High peaks are observed clearly at separation distances that correspond to the ordered packing, for *Φ* = 60%. But other ones appear at unexpected separation distances. Thus, the spatial arrangement of the covered microemulsions contains both cristal-like and disordered structures. This structure is in a glassy state, as reported in previous studies.^37^ When *Φ* increases significantly, the van der Waals attraction dominates, and the repulsive interaction diminishes. Thus, the O/W-MIS approach closely each other. Then, clusters are formed, and the interconnection between them increases with *Φ*, from sol to gel and from gel to crystal-like states.

### 3.2 Effect of added (D–PEO_227_–D) on the microdistribution of decane/water microemulsions

**Fig. 6:**
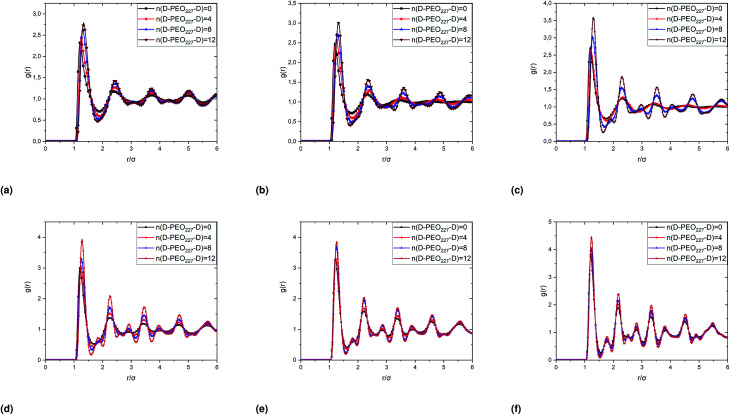
Pair correlation function *g*(*r*) of the microemulsions with different added *n*(D–PEO_227_–D), obtained from the MD simulations, plotted as a function of the volume fractions corresponding to the phase transitions. *Φ* = 26.5% (a), *Φ* = 29% (b), *Φ* = 31.5% (c), *Φ* = 33.5% (d), *Φ* = 35% (e), *Φ* = 37% (f).

In Fig. 6, we report the *g*(*r*) as a function of the separation distance between the covered microemulsions (*r*) for several values of *n*(D–PEO_227_–D) per microemulsion using MD simulation. We notice that at short separation distances from the size of the microemulsions hydrophobic part, *i.e.*, for 0 < *r* < 2*R*, RDF practically goes to zero due to the strong electrostatic repulsion between two adjacent microemulsions. These results obtained for bare microemulsions also confirm that the microemulsions' shape and size remain unchanged. Then, in this distance regime, there is a correlation hole. These pronouncements are independent of the microemulsions volume fraction, neither the added polymers per microemulsions *n*(D–PEO_227_–D), or temperature.

For low volume fractions, corresponding to a sol state, over distances of the order of a few microemulsions size. The *g*(*r*) mains peaks have a Gaussian-like form. Thus, the microemulsions that present a normal-probability distribution around a considered central one are weakly correlated. We note that the standard deviation from the most probable separation distance depends crucially on the microemulsions volume fraction and the added polymers per microemulsions *n*(D–PEO_227_–D). As the volume fraction increase, secondary peaks are observed, and the standard deviation at the first peak diminishes. Thus, the microemulsions show an ordered liquid distribution. In reverse, when *n*(D–PEO_227_–D) increase the principal peak-height shift toward the right, and the standard deviation increases. In this case, the separation distance between adjacent microemulsions increases, and they are disordered in space. This disorder is due to the repulsion interaction induced by the presence of a high amount of polymers (*U*_steric_), which is in addition to the electrostatic repulsion overcomes the vDW attraction and the bridging effect. Thus, for moderate volume fractions, the polymers contribute by a repulsive interaction rather than the attractive one. At very long distances, *g*(*r*) practically tend to 1, regardless of the amount of added *n*(D–PEO_227_–D).

For high volume fractions, *g*(*r*) oscillates and appears as a succession of peaks of decreasing heights. Thus, each microemulsion is surrounded by numerous neighboring shells. The locations of the maxima of these oscillations correspond to the preferred distances between neighboring microemulsions. Between these maxima, there are localized regions of relatively low density. As shown in this figure, as the *n*(D–PEO_227_–D) is increased, the *g*(*r*) first peak position, *R*_max_, shifts slightly to the right. This shift is due to the polymers steric repulsion. Fig. 6 also shows that the main peak height is increased by an increase in *n*(D–PEO_227_–D). Such an increase can be attributed to the attraction caused by the bridging effect. This attraction acts at specific separation distances 2*R* + *R*_g_ < *r* < 2*R* + *R*_F_. When the volume fraction of the microemulsions increases, a gradual decrease in the repulsive part of the overall interaction potential, gives rise to an aggregation of the covered decane/water microemulsions in saltwater.

**Fig. 7:**
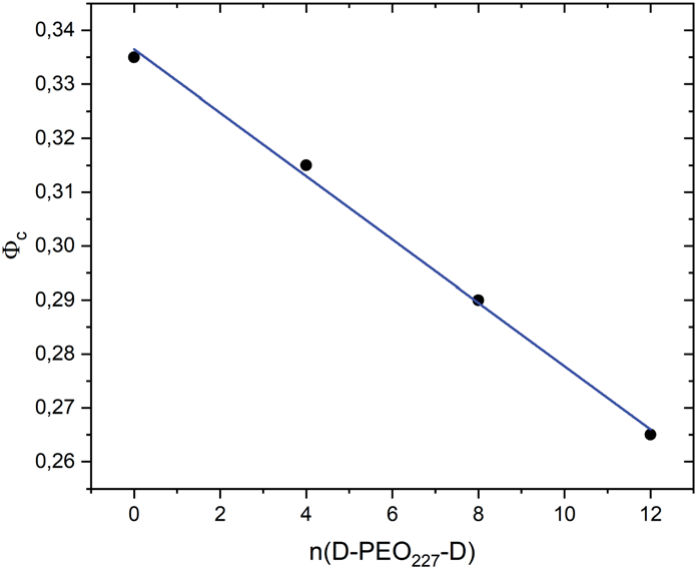
The dependence of the critical volume fraction *Φ*_c_ on the number of added polymers *n*(D–PEO_227_–D).

Fig. 7 show the dependence of the critical volume fraction *Φ*_c_, correspond to the transition sol–gel, on the number of added polymers *n*(D–PEO_227_–D), summarized from the analysis of the *g*(*r*) curves calculated from MD simulations as presented in Fig. 6. Accordingly, *Φ*_c_ decreases linearly as a function of the parameter *n*(D–PEO_227_–D),

12 *Φ*_c_ = *b* + *a* × *n*(D–PEO_227_–D)

with the slopes *a* = −0.59 × 10^−2^ ± 0.2 × 10^−3^, and *b* = 33.65% ± 0.1% the *Φ*_c_ of uncoated microemulsions *n*(D–PEO_227_–D) = 0.

This decrease in the *Φ*_c_ means that the correlations between the microemulsions, which cause the structural arrest, increase with the presence of two hydrophobic extremities polymer chains grafted to the MIs surfaces. The polymers bridge the microemulsions and form a spanning network. This effect is strongly proportional to the volume fraction.

**Fig. 8:**
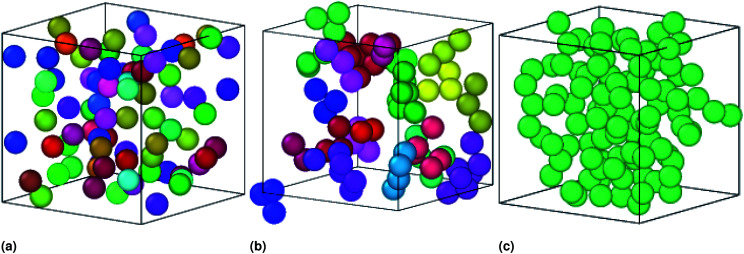
Sample captions of the simulated microemulsions showing the effect of the added polymers on the microemulsions arrangement at room temperature, using the Open Visualization Tool (OVITO) (https://www.ovito.org). (a) Simulation of a volume fraction *Φ* = 22% of uncoated O/W-MIs, (b) simulation of a volume fraction *Φ* = 22% O/W-MIs coated with 12(D–PEO_227_–D), (c) simulation of a volume fraction *Φ* = 31% of O/W-MIs coated with 12(D–PEO_227_–D) polymers. The number of O/W-MIs used to produce the structural properties is *N* = 10^6^. Each color corresponds to a cluster of an ensemble of connected particles, which is nonconnected with the others. The particles represented in various colors belong to different aggregates (Fig. 8a and b) miss a continuous path, originated from the attractive interaction dominance, connecting them to create a spanning network (Fig. 8c) because of the weak attraction between them. The number of particles belongs to each cluster increase with increasing *Φ*, increasing *n*(D–PEO_227_–D) per microemulsions, or decreasing the temperature (for clarity the simulation is performed for few numbers of microemulsions).

In similar works using SANS, dynamic light scattering (DLS), and fluorescence correlation spectroscopy (FCS) measurements, Paula Malo de Molina *et al.* studied the effect of a doubly hydrophobic terminated water-soluble polymer (C_18_H_37_–PEO_150_–*C*_18_H_37_) on the properties of an oil-in-water (O/W) microemulsions. They showed, from SANS measurements, that the size of microemulsions is not affected by polymers addition (as we confirmed in Fig. 2). At low polymer concentration, the polymer can bridge some microemulsions. At intermediate polymer concentration, the bridging effect increases resulting in the formation of clusters of interconnected particles. Also, they showed that, at higher polymer concentrations, the microemulsions are attached and forming a spanning network,^51^ in good agreement with our results as presented in Fig. 8.

### 3.3 Effect of temperature on the microdistribution of decane/water microemulsions

**Fig. 9:**
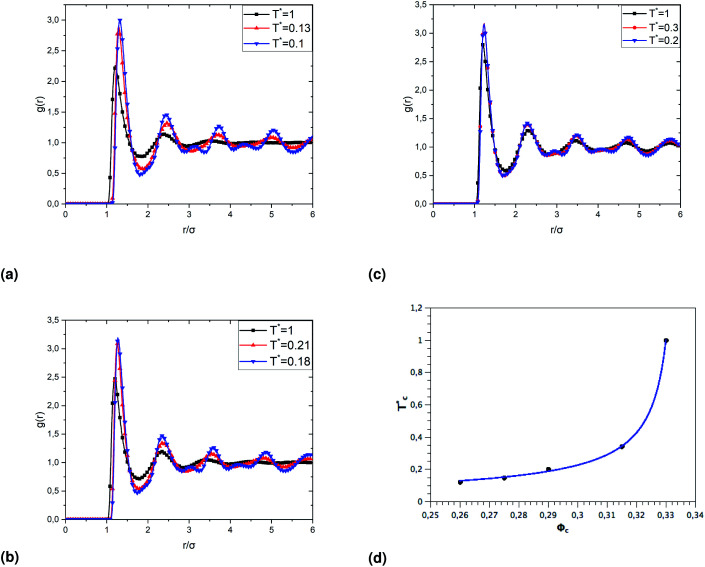
The dependence of the critical temperature 
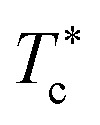
 on the volume fraction *Φ*_c_ of uncoated MIs, obtained from the analysis of the *g*(*r*) curves calculated from MD simulations.

To investigate the sol/gel transition, controlled only by decreasing temperature, we simulate low volume fractions of uncoated microemulsions (*Φ*_c_) supposed to be in the sol state for room temperature. Fig. 9 shows the calculated sol/gel transition temperature of uncoated O/W-MIS as a function of the volume fraction. By decreasing temperature, the *g*(*r*) curves narrow and increase in high, the minima peaks split into two adjacent minima, and a secondary maxima peak appears between them. This change in the *g*(*r*) curves can be explained by the fact that the low volume fraction microemulsions lost their fluid characters and form gel structures. As observed in similar published studies,^52–54^ the critical temperature, corresponding to the loss of the sol state characteristics, shows a pronounced increase with increasing *Φ*,13
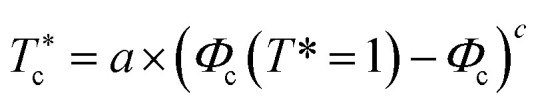
with the slope *a* = 0.02 ± 0.004, the critical volume fraction at the room temperature *Φ*(T* = 1) = 33.45% ± 0.1%, and the exponent *c* = −0.73 ± 0.07.

**Fig. 10:**
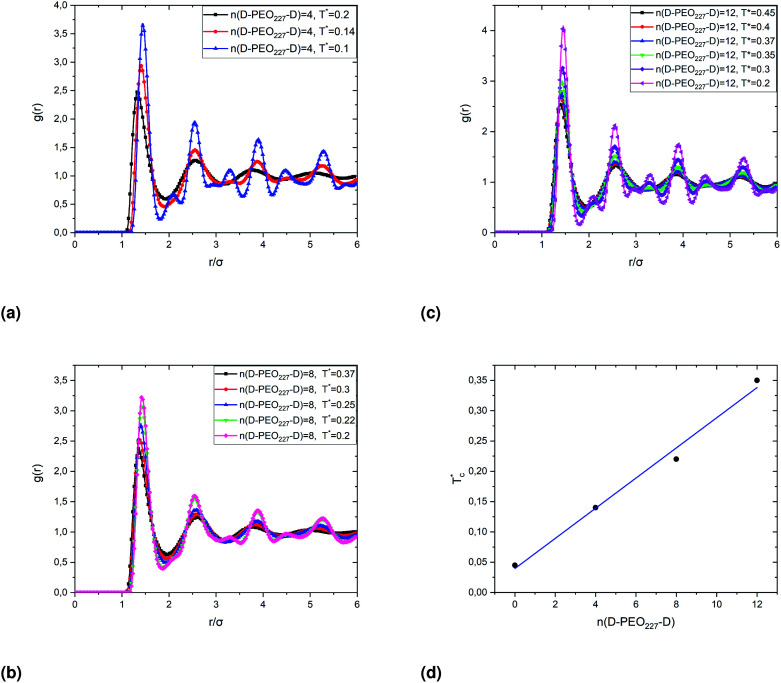
The dependence of the critical temperature 
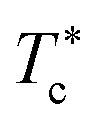
 on the added polymers *n*(D–PEO_227_–D) for *Φ* = 23%, obtained from the analysis of the *g*(*r*) curves calculated from MD simulations.

The radial-distribution-function *g*(*r*) results of covered microemulsions in the first gelation process, for *Φ* = 23%, are shown in Fig. 10a–c. Under the ambient temperature T* = 1, the *g*(*r*) shows regulate structures, which indicates they maintain the sol state within the temperature range above the critical one 
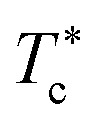
. For temperatures below 
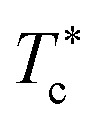
 the *g*(*r*) curves narrow, and every single minimum peak split into two adjacent minima, and a maximum one appears between them, which indicate the sol has lost its fluid characters and enters into a state of gel structures.

When the temperature is low 
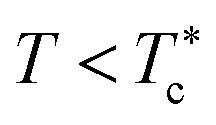
, the maximum peaks characteristic of the *g*(*r*) plots are apparent compared to those obtained for the temperature above 
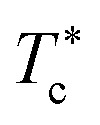
, which characterizes the strong correlation between the microemulsions. The increase in temperature causes the height of the peaks to decrease periodically or disappear. This disappearance of the *g*(*r*) peaks is a symbol of the sol state characterized by a weak correlation and well dispersion. As the number of graft polymers increases for fixed volume fraction values, the transition sol/gel occurred swiftly by decreasing the temperature. The maximum peaks of *g*(*r*) become intense also, the number of *g*(*r*) maxima increases and maintain the first peak sharp and apparent. After the gelation temperature 
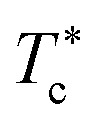
, the *g*(*r*) peaks persist in decreasing slowly for covered microemulsions compared to bare microemulsions. Simultaneously, the curves disappeared imperceptibly in the region far from the center, proportionally to the number of added polymers per microemulsion. According to the above analysis, Fig. 10d shows that 
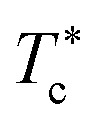
 increases linearly with increasing *n*(D–PEO_c_–D),14
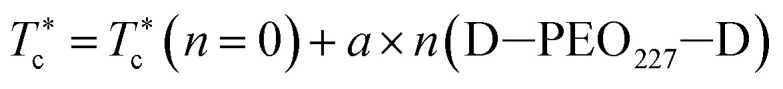
with the slope *a* = 0.025 ± 0.001, and 
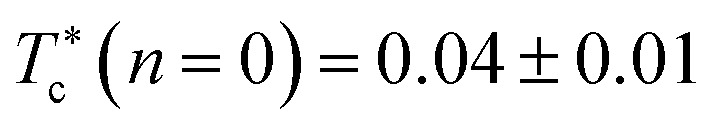
 the gelation temperature of uncoated microemulsions *n*(D–PEO_227_–D) = 0.

We note that when the temperature is below 
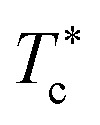
, a tiny peak is observed in the middle of each minimum. The first maximum peak represents the broadest correlation between microemulsions in the system, mainly observed for the minimum approach distance between particles. The tiny peak could be related to the microemulsions arrangement characterized by fewer coordination numbers where the surrounding microemulsions occupy a high-energy area of space. Besides, this peak is also the first to disappear if the temperature exceeds 
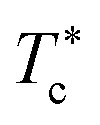
.

## 4 Discussions

At living temperature, uncoated decane/water microemulsions sol/gel transition occurs at a volume fraction, *Φ* = 33.65%. Then, by increasing *Φ*, a glass transition starts at *Φ* = 37% and pronounces at *Φ* = 60%. Besides, when decreasing the temperature at high *Φ*, the effect of van der Waals attraction leads to a sol/glass transition. At freezing temperatures and high *Φ*, the microemulsions distribution shows clusters formation and structural arrest with a gel characterized by connected chains-like structures.

We note that an extensive increase of the volume fraction leads the O/W-MIs to form a bicontinuous structure.^55^ The bicontinuous structure is a homogeneous lipophilic and hydrophilic equilibrium phase.^56,57^ In this type of structure, both the oil phase and the water phase are continuous. The junction of the water and oil phases is represented by a monolayer of the surfactant molecules, which can spontaneously arrange themselves into a structure with zero or low curvature on average.^58^ Thus, using cover polymers that bridge the O/W-MIs is a matching alternative to increasing the space correlation between O/W-MIs.

For coated O/W-MIs, the space distribution is surprisingly characterized by equilibrated cluster formation and structural arrest, even at low temperature and low *Φ*. The addition of the D–PEO_227_–D triblock copolymers to the decane/water microemulsions has two contributions in its interaction potential, repulsive and attractive, which depend on *Φ* and *n*(D–PEO_227_–D). By increasing *Φ* or *n*(D–PEO_227_–D), analysis of the O/W-MIs distribution revealed that the bridging effect increase. Thus, the added polymers form effective bonds in the microemulsions gel network. In contrast, for low *Φ*, bonding the two hydrophobic parts to the same microemulsions or between few neighboring particles is favored. Thus, equilibrated clusters are formed instead of percolation gel. These confirmations are in good agreement with recently published works.^33,51^

At the critical temperature 
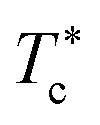
, the particles form clusters due to the attractive interactions that favor the aggregation. The attractive interactions include the van der Waals attraction and the attraction induced by the graft polymers bridging effect.

However, the bonds between the microemulsions are strong, in the case of gelation, by the bridging effect, then by van der Waals attraction. In any event, it is expected that at freezing temperatures, the correlation between microemulsions will be extremely high, and the appearance of a spanning network could produce a phenomenon closely related to standard irreversible gelation. Fig. 6, 10 and Table 2 show that the mean distance between the microemulsions increase by increasing *n*(D–PEO_227_–D) or by decreasing the temperature. Also, this distance is longer for the gel corresponds to the structural arrest, compared to that for the gel obtained by the bridging effect. At a higher temperature, the formation of the bonds decreases. However, as the volume fraction increases, the added polymers could eventually cause a structural arrest.

The dependence of the minimum approach distance between microemulsions on *T* and *n*(D–PEO_227_–D)

**Table:** 

*Φ* = 31%						
T*	1	0.5	0.4	0.3	0.2	0.1
*r*_max_	1.19	1.22	1.23	1.235	1.27	1.30
*g*_max_	2.70	2.89	3.00	3.12	3.56	4.32

**Table:** 

*Φ* = 31%				
*n*(D–PEO_227_–D)	0	4	8	12
*r*_max_	1.19	1.21	1.28	1.30
*g*_max_	2.70	2.65	3.05	3.59

Percolated gel occurs at a low fraction, while for a higher volume fraction, the structural arrest is observed even at elevated temperatures. At high temperatures and low *Φ*, the microemulsions are well dispersed. If the fraction increased gradually, equilibrated clusters are formed. At freezing temperatures, the percolated chains and structural arrest have the same behavior. In, therefore, the structural arrest is expected to combine many gelation and glass transition characteristics.

Generally, the gelation behaviors of colloidal particles are decided by the change in one of the following three keys parameters: the temperature, the volume fraction, and the nature of the interactions that can be controlled by surface graft polymers. In this work graft polymers induce an attractive interaction resulting from the bridging effect.

## 5 Conclusion

This study aims to examine the interaction and structural behavior of oil/water microemulsions (O/W-MIs) covered by PEO_227_ polymer chains modified by hydrophobic alkyl (dodecyl) in its two extremities that we noted (D–PEO_227_–D), using an MD simulation method. A particular emphasis is focused on the sol/gel transition that occurred by controlling the volume fraction, the number of added polymers per microemulsion, or the temperature. The determination of the form and size of the studied microemulsions and the validation of the potential parameters, which reproduce the O/W-MIs physical reality, are guided by SANS experiments. The addition of a different number of *n*(D–PEO_227_–D) chains per microemulsions, performed by replacing a small amount of surfactant and cosurfactant with the appropriate *n*(D–PEO_227_–D), conserve the O/W-MIs shape and size. Besides, the sol/gel volume fraction decreases linearly, and the gelation temperature increases linearly with increasing *n*(D–PEO_227_–D).

Considering the O/W-MIs physicochemical properties, the sol/gel transition process by temperature decrease or increasing the volume fraction is not always a practical choice. Also, the increase in the volume fraction demands additional quantities of oil, surfactant, and cosurfactant. Thus, two ends hydrophobically modified polymers are a privileged solution to use O/W-MIs in the gel state at living temperatures.

## Conflicts of interest

There are no conflicts to declare.

## Supplementary Material
